# Enabling the Orchestration of IoT Slices through Edge and Cloud Microservice Platforms

**DOI:** 10.3390/s19132980

**Published:** 2019-07-05

**Authors:** Juan-Manuel Fernandez, Ivan Vidal, Francisco Valera

**Affiliations:** 1 Research and Development Department, Ericsson Spain S.A., Vía de los Poblados 13, 28033 Madrid, Spain; 2 Departamento de Ingeniería Telemática, Universidad Carlos III de Madrid, Avda. Universidad, 30, Leganés, 28911 Madrid, Spain

**Keywords:** Internet of Things (IoT), network slicing, orchestration, edge computing, microservice, Small Unmanned Aerial Vehicle (SUAV), IoT slice

## Abstract

This article addresses one of the main challenges related to the practical deployment of Internet of Things (IoT) solutions: the coordinated operation of entities at different infrastructures to support the automated orchestration of end-to-end Internet of Things services. This idea is referred to as “Internet of Things slicing” and is based on the network slicing concept already defined for the Fifth Generation (5G) of mobile networks. In this context, we present the architectural design of a slice orchestrator addressing the aforementioned challenge, based on well-known standard technologies and protocols. The proposed solution is able to integrate existing technologies, like cloud computing, with other more recent technologies like edge computing and network slicing. In addition, a functional prototype of the proposed orchestrator has been implemented, using open-source software and microservice platforms. As a first step to prove the practical feasibility of our solution, the implementation of the orchestrator considers cloud and edge domains. The validation results obtained from the prototype prove the feasibility of the solution from a functional perspective, verifying its capacity to deploy Internet of Things related functions even on resource constrained platforms. This approach enables new application models where these Internet of Things related functions can be onboarded on small unmanned aerial vehicles, offering a flexible and cost-effective solution to deploy these functions at the network edge. In addition, this proposal can also be used on commercial cloud platforms, like the Google Compute Engine, showing that it can take advantage of the benefits of edge and cloud computing respectively.

## 1. Introduction

According to the IoT Analytics market update published in August 2018 [1], the number of Internet of Things (IoT) connected devices by the end of 2018 was expected to be around 7 million, and the expected number of IoT devices in 2025 would be around 21.5 million, exceeding the number of non-IoT devices.

Forbes [2] estimates that the revenue growth in IoT will double by 2025 compared to 2017 revenues, endorsing the idea of how promising the IoT market will be in the coming future. However, even though expectations are still high, the number of IoT implementations previously foreseen for 2020 are below initial prospects. Following Forbes, the main barriers for IoT adoption are “security, integration with existing technology and uncertain returns on investment”.

Regarding the integration barrier, one of the main challenges to be faced by IoT technologies is related to the big heterogeneity [3] of the IoT world. This heterogeneity is associated with the variety of use cases where these technologies can be applied, the many different types of devices, the diverse connectivity mechanisms supported (especially wireless) and the number of protocols and IoT platforms. Interoperability and integration are key requirements for these existing solutions, not only from the IoT platform perspective, but also from the network side. Thus, 5G networks, which are today under development, are designed and planned to cover IoT heterogeneity.

In relation to this variety of use cases introduced by IoT, a relevant characteristic of the existing commercial IoT platforms is the potential latency introduced by their main components executed in the cloud. For this reason, it is crucial to offer the possibility to execute distinct IoT functions (e.g., gateways, databases, or analytics servers) in different locations of the network, depending on the requirements of the use case under consideration. For instance, an IoT gateway may be deployed at an edge domain, to enable a prompt inspection and transformation of the high-volume data that are typically generated by IoT devices, performing computational tasks in locations that are close to the devices themselves. Other functions, like databases and IoT analytics servers may run in a cloud domain, converting data received from the edge computing platforms and IoT devices into formats that enable a more convenient storage of these data, and analyzing these data with no real-time constraints. Some of the existing commercial platforms provide the capability to run some components at the edge, but still some specific network support is required. The flexibility provided by 5G networks [4] is vital to support edge computing.

One important aspect to be considered about the flexibility offered by 5G networks is the possibility to share network resources among distinct IoT solutions or even with other applications. The existing IoT platforms are already supporting multi-tenancy, allowing several customers to share the same computing resources, applications or even services in an isolated way. However, the existing networks have several limitations isolating traffic, something which is mandatory to allow the cohabitation of different types of use cases. Therefore, 5G offers the possibility to have isolated partitions of the whole network enabling traffic segregation by the definition of network slices.

The Global System for Mobile communications Association (GSMA) [3], one of the most relevant organizations representing the mobile network operator industry, defines a network slice as an “independent end-to-end logical network that runs on a shared physical infrastructure, capable of providing an agreed service quality” and it can be used “to serve a defined business purpose of a customer”.

### 1.1. Problem Statement

Apparently, all the necessary components to provide interoperability in the IoT arena are already present: IoT platforms are supporting multiple customers on the same infrastructure (i.e. multi-tenancy), networks are supporting network slicing, it is possible to deploy functions and applications in different network locations, and lastly, IoT platforms and networks are supporting multiple protocols and types of traffic. However, there is still a key area in the IoT industry where the required interoperability is not providing a complete solution: the automation and orchestration. The components responsible of this orchestration must be able to configure and deploy the network slices and IoT functions (e.g., an IoT gateway) in the proper locations depending on the use case.

By putting the network slices altogether with the IoT platform multi-tenancy, it is possible to define the IoT slice concept. An IoT slice would then be a partition of the whole end-to-end IoT solution assigned to a specific customer or most probably to a group of customers requiring similar use cases. The orchestration and integration area must embrace this IoT slicing concept.

Despite of the flexibility provided by 5G networks, they are still not flexible enough to support some specific devices and protocols used in IoT solutions (e.g., ZigBee [5] or 6LowPAN [6]). In these situations, IoT gateways responsible for translating protocols at different levels will still be needed. These IoT gateways can also be software based, allowing them to be deployed into fog or edge nodes with eventual resource constraints (energy or computational). For instance, a particularly interesting platform to support gateway functions are Small Unmanned Aerial Vehicles (SUAV), which can be flexibly deployed at the network edge to serve to a variety of use cases.

### 1.2. Objectives

The main target of this article is to address the barrier highlighted by Forbes [2], regarding the integration of IoT solutions with existing technologies like cloud computing and platform multi-tenancy, but also with other newer technologies like edge computing or network slicing, as defined for 5G networks. This important integration part is related to the deployment, configuration and orchestration of the diverse components available in the IoT ecosystem into isolated end-to-end IoT slices.

The article is particularly focused on the automation and orchestration aspects, introducing the architectural design of an IoT slice orchestrator. This design is compatible with the network slicing concept and intended to cover a wide range of IoT use cases. Furthermore, as a first step to prove the practical feasibility of our solution, the article presents a prototype implementation of this orchestrator which is able to deploy IoT functions (IoT gateways and IoT servers) in different locations of the network (edge and cloud) for a given slice. In order to have a realistic environment, several types of infrastructures are used for the functional validation of the prototype, including constrained computing nodes like Single Board Computers (SBCs) that can be found at edge domains and commercial cloud computing platforms like Google Compute Engine (GCE) [7].

With the development and validation of this prototype the following objectives are achieved:

- Prove that it is possible to coordinate, in a real scenario, the deployment of functions composing a multi-tenant (sliced) IoT solution, despite its heterogeneity.
- Verify that the orchestration can be done through different locations and is hence compatible with edge and cloud computing, taking advantage of their benefits, i.e., the low latency provided by edge computing whenever quick response times are required, and the flexible and automated allocation of resources supported by cloud computing technologies.
- Demonstrate that it is possible to perform this orchestration even with constrained devices where IoT functions can be executed.
- Confirm that lightweight virtualization techniques are propitious to achieve these purposes.

The rest of this article is organized as follows: Section 2 reviews the existing literature and ongoing research on the usage of edge computing, microservice architectures and orchestration in IoT solutions. The architectural design of the proposed solution is included in Section 3. Section 4 includes details about the provided prototype and the results obtained from its validation. Finally, Section 5 concludes this article and provides some future research lines.

## 2. Background and Related Work

This section will cover previous research that has been used as the main reference work for this article, and that is related to the topics of our research (edge and fog computing, microservice architectures, network slicing and end-to-end coordination of resources in IoT solutions).

### 2.1. Cloud, Edge and Fog Computing

Cloud computing techniques are extensively used in many different sectors, including the IoT segment where the multi-tenancy support of this technology is particularly important. However, as it was presented in Section 1, cloud computing may not be the most appropriate approach in use cases that require the processing of data with rapid response-times. In these situations, edge or fog computing (even combined with cloud computing) could be used instead.

There has been a significant effort in the research and industry communities to develop edge and fog computing technologies. Sometimes those terms are applied indistinctly to certain nodes in the access network (e.g., small central office data centers), to computational resources allocated in antennas or base stations, or even to computational and storage resources in devices, routers or gateways connected to the access network. In any case, both terms are referring to resource platforms that enable the execution of applications, or parts of applications, and are much closer to the end-user devices than the core data centers used in cloud computing, achieving much lower latencies. The surveys provided in [8,9] offer a complete and useful comparison of edge and fog computing models and a classification of fog computing research areas that are relevant to understand these concepts. In addition, the architecture proposal included in [10,11] presents a three-layer classification of cloud, edge and fog computing techniques based on the latency required by the use case and the amount of generated data.

In general, it is possible to differentiate two edge scenarios: network edge and device edge (fog and mist levels [10]). The first case makes use of nodes at the edge of the network with sufficient computing and storage resources so as to be able to execute almost any type of load, like small office data centers or even resources in antennas or base stations; while the latter is more heterogeneous and makes use of the scarce computing and storage resources available in the devices themselves. Even though network edge scenarios might probably be more widely deployed, some use cases (like the ones using SUAVs) will certainly make use of device resources. As a related work, a detailed proposal about how to address the optimal placement of network functions at the edge can be found in Akraino’s edge stack documentation [12].

The concepts of fog and edge computing are particularly applicable to IoT solutions. In this respect, the reference model for IoT deployments defined by the IoT World Forum (IoTWF) [13], identifies fog and edge computing as fundamental technologies to enable the analysis and transformation of the high-volume of data that are typically generated by IoT devices, moving computational tasks to locations that are close to the devices themselves.

Going into detail, fog and edge technologies may provide the following value-added features:

- Much better latency figures in solutions where a fast reaction is needed, like connected cars or autonomous vehicle use cases, since the computation is performed much closer to the device.
- Data can be processed at the edge, allowing it to send aggregated data, reducing overheads and redundant information and using bandwidth much more efficiently.
- Better utilization of resources. Nowadays, most edge nodes are not using all the available computation resources during their regular tasks (e.g., a home router might not be using all its resources to route packages to the Internet).

In addition, the IoTWF IoT reference model [13] emphasizes the relevant role that cloud computing platforms may have in the IoT, providing data accumulation capabilities that offer convenient mechanisms to process and store of these data, such that they can be available on demand to interested IoT applications.

However, in the scope of the IoT, the existing work on fog, edge and cloud computing platforms does not consider the automated and coordinated allocation of functions or applications over those platforms. This concept, referred to as service orchestration, is fundamental to building functional and isolated end-to-end IoT applications, leveraging the aforementioned advantages of edge and fog computing solutions.

According to Suárez-Albela et al [14], most IoT solutions contain IoT nodes (devices) that are collecting information from the environment, applications deployed in the cloud that are processing this information (e.g., analytics applications) and IoT gateways that are connecting the devices with an IoT application running in the cloud. These IoT gateways are normally allocated to the edge, but its deployment in the cloud is not precluded.

### 2.2. Microservice Based Architectures in IoT Environments

Software virtualization has possibly been one of the most important technological improvements of the last decades in software engineering, since it allows the execution of any type of load in centralized data-centers, offering scalability and elasticity to the system, requiring smaller and more efficient hardware (using only what it is really needed) and hence reducing both operational and capital expenditures. However, classical virtualization techniques (based on the utilization of hypervisors [15]) do not seem to fit very well in edge and fog computing environments due to their significant resource requirements that might not be available in edge nodes due to their typical capacity limitations.

Nevertheless, lightweight virtualization based on containers or even unikernels [16] does not introduce such strong computing requirements, allowing the execution of small loads on any constrained device such as single board computers. Microservice-based architectures, where an application is decomposed in “small services”, are making use of this lightweight virtualization, with each microservice deployed as one or several containers. This results in the implementation of different parts of an application in an independent way, but also allows the execution of these parts in multiple nodes which can communicate using specific well-defined interfaces, something that was not possible in monolithic architectures.

These architectures are very convenient for applications that can benefit from edge and fog computing, like IoT solutions [17], where parts of them can reside in the cloud (the ones with higher computational requirements and less dependency on latency) or at the edge (the ones with strong latency requirements). The capillary computing architecture included in [18] proposes the usage of microservices in a Vehicle to Everything (V2X) solution in order to dynamically offload traffic from cloud computing servers to edge and fog nodes depending on some parameters, like resource usage (e.g., CPU load) or network related parameters (e.g., latency or jitter).

Even though dynamic traffic offload towards the edge is a good example of benefits from edge and fog computing in IoT solutions, those articles are not addressing the problem targeted in this article: the deployment and configuration of applications composing an IoT solution, in different locations, and based on different customer (tenant) requirements. This would be the case, for instance, of an IoT smart farming solution integrating two services, one to control the humidity in some vineyards and another one to manage autonomous UAVs operations used for cattle surveillance. In the first use case there are not important latency requirements and hence everything can be deployed in the cloud infrastructure. In the second use case there are significant latency requirements and some parts of the solution may initially be deployed in the cloud while some others at the edge (e.g., in base stations or even in the UAVs themselves).

Using microservice architectures is not just a software design choice, since the implications in their deployment are remarkable. Each small service has its own availability, capacity and scalability requirements, but also its own lifecycle management. These requirements and management characteristics must be coordinated (orchestrated) throughout the network, so that the complete solution built by these microservices does also provide the right availability, capacity and scalability requirements together with the proper configuration. Implementing all these tasks manually or using basic tools like Docker Compose [19] can be extremely hard, and there are other container orchestrators that can be used to facilitate these procedures (Kubernetes [20], Docker Swarm [21] on top of Docker Compose, Marathon [22] or Nomad [23]). Alam [24], Hegyi [25] et al propose for instance the usage of Docker Swarm to orchestrate IoT applications based on microservices, but not in a coordinated way. The main intention of these articles is to prove the benefits brought by microservice architectures to the IoT world, and they consider that there is only a unique Docker Swarm infrastructure distributed along different parts of the network using multiple types of hardware. Although this is an interesting topic that is currently under investigation in the IoT industry [26,27,28], it is not very realistic today to have a single managed platform running on top of heterogeneous hardware (like powerful computers in the cloud and constrained SBCs at the edge) and connected through different networks with their own quality of service. Platforms can be heterogeneous in the IoT arena and this paper ambition is to introduce an orchestrator function that is able to handle this diversity.

### 2.3. Network Slicing in IoT

Most IoT solutions already allow IoT applications and services sharing among several customers (or tenants) and also physical resources sharing. This means that multiple tenants can share the same platform infrastructure (i.e., computational, network and storage resources within a data center) or even the IoT devices sending measurements collected from sensors. There is considerable literature about those topics, like the Platform as a Service (PaaS) framework proposed by Li et al in [29], the possibility of sharing IoT devices described by Benazzouz et al in [30] and the multi-tenancy decentralized architecture included by Cherrier et al in [31]. Regarding IoT application and service sharing, the microservice concept has this multi-tenancy requirements embedded as one of its key features, allowing it to deploy dedicated and independent microservices for each tenant. Hence, any microservice-based application is in fact multi-tenant, per se.

However, network slicing [32,33] goes beyond these multi-tenancy bases, since it allows the partitioning of the networks connecting the locations and data centers where virtualized network functions are installed and avoiding any interference between functions allocated to different partitions. That is, the access [34,35], transport and core networks can be split into independent logical networks that can be assigned in turn to different customers. Network slicing is one of the key concepts introduced by 5G networks [36] which are designed and intended to provide this type of partitioning.

**Figure 1:**
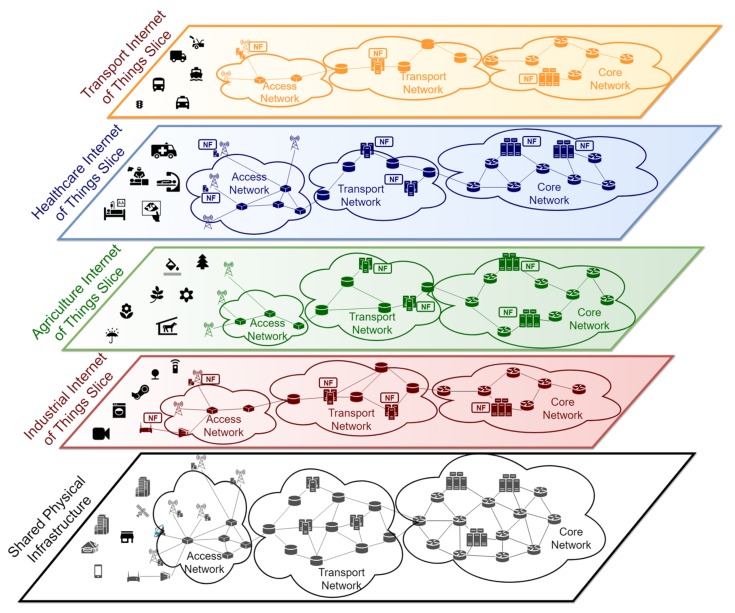
Internet of Things (IoT) network slicing example.

Figure 1 shows an example with several IoT slices allocated to different verticals. These verticals share the same physical infrastructure, composed by: an access network (wireless and/or wireline), a transport network, and a core network. The core network hosts the equipment with higher capacity, while more constrained computing devices are installed close to the IoT devices (i.e., sensors and actuators). From the vertical’s perspective, now there is some isolated network infrastructure allocated to them, infrastructure which provides specific functions and performance figures:

- The industrial IoT slice is used, for instance, to connect robots and other devices to some applications with access to the network. This kind of services typically needs low latency communication, requiring the deployment of functions at the edge of the network (e.g., access network)
- The agriculture IoT slice might be used to monitor and deliver relevant information in smart farming applications, e.g., values of temperature or humidity in different regions of a crop field. In this case, functions able to process high volumes of data may be required in nodes allocated in cloud computing infrastructures that are close to the core network.
- The IoT slice devoted to the healthcare market segment may have devices requiring low latency communications, as well as application processes with high capacity demands, both in terms of computation and storage, which may be satisfied by a cloud computing infrastructure.
- Finally, the IoT slice for intelligent transportation systems may connect autonomous cars, as well as other types of connected vehicles, to automotive-specific applications. These applications may require the execution of specific functions at the edge, in order to guarantee the execution and actions with strict delay constraints, and/or at the cloud, to support non-sensitive computation tasks and the permanent storage of automotive-related data (e.g., maps, route calculations, etc.)

In order to accomplish this network slicing, technologies like Network Function Virtualization (NFV) or Software Define Networking (SDN) are used. These technologies, which are considered as fundamental building blocks of the new 5G networks, are also applicable to the IoT arena. Some examples about the usage of SDN and fog computing in IoT [37] and the orchestration of the connectivity between several IoT functions [38,39] can be found in the existing literature.

By putting together multi-tenant applications with network slicing, it is possible to have dedicated and independent IoT business solutions for specific verticals, deployed on the same infrastructure and using the same underlying networks. This multi-tenancy is an important requirement for many IoT applications like augmented reality, smart sensor networks or big data analytics as it is already highlighted by Morabito et al in [16].

Network slicing introduces a high degree of flexibility to support devices with different requirements in terms of quality of service, as well as to optimize the network usage, according to IoTWF [4] and Afolabi et al [40]. This flexibility is achieved with the help of virtualization techniques by providing independent networks over a shared infrastructure. Each one of these networks is used for distinct business purposes. However, this implies greater complexity for the network owner’s operation departments since it is yet another aspect to be configured and maintained. This complexity requires the introduction of a network slice orchestrator responsible for considering all the business and technical provisioning requirements.

The orchestration of network slices is still at an early stage. However, the IoT slice resource orchestration included in [41] describes the possibility for the operators to define network slicing as a Service (NSaaS) and propose the introduction of an intelligent 5G network slice broker. This broker makes use of the information provided by IoT gateways to optimally reconfigure and provision network slices. Another proposal to orchestrate and federate network slices for vertical players is the 5G Transformer orchestrator described in [42]. Some other examples of network slicing orchestration as the one described by Tosi et al [43] are still in a very initial phase.

This article considers multi-tenancy aspects at the edge and the cloud domains along with network slicing at the different transport networks involved in the device-to-edge and end-to-cloud communications. This enables a holistic view of the slicing concept applied to an IoT solution, where our IoT slice orchestrator is capable of providing the required resources (computation, networking, and storage) across all the different domains involved in the execution of an IoT service. However, although the system is designed for this purpose, the prototype implementation is mainly focused on the multi-tenancy aspects at the edge and the cloud, leaving the network slicing part for a future development, due to its complexity and the lack of maturity of network slicing solutions specially in the open source ecosystem.

### 2.4. End-to-end Coordination of IoT Resources

There are not many previous studies providing a holistic view of an end-to-end IoT application, mainly as a result of the vast heterogeneity of IoT solutions. This heterogeneity starts from the huge variety of use cases they can address (e.g., from health care to smart cities for instance) and hence the requirements they might cover (e.g., latency, persistence, storage capacity, analytics). In addition, the different technological aspects associated to IoT (e.g., type of devices, connectivity means, IoT platforms) and the variety of standardization bodies trying to address all these aspects (e.g., ETSI, oneM2M [44] or IOTI [45] among others) must also be considered.

Le [46], Truong [47] et al succeeded on providing this global view, considering physical resources (IoT devices mainly) and virtual resources allocated at the edge (what they call “IoT sites”) where IoT gateways are commonly (but not compulsorily) deployed, also inside the network as Virtual Network Functions (VNFs) and finally inside the cloud data centers where IoT cloud services are placed. Thus, authors define the concept of an interoperability hub, so that, regardless of what it is finally used in the implementation, a similar view of the solution is provided to the operator. Hence, the main purpose of this interesting analysis is to harmonize the view of multiple IoT solutions, considering an important variety of real implementations which could be used at the edge, the transport network and the cloud. Still, it does not cover how these functions are deployed and how they can be organized into slices.

The SINC project [47] also provides an information centric view of a global IoT solution and there is a proposal from Nguyen et al [48] of how to use software defined networking techniques to connect the functions composing an end-to-end IoT solution. However, they are not covering the coordination of the deployment of the pieces composing an IoT solution into IoT slices. In other papers, it is also possible to appreciate different approaches aiming at coordinating IoT resources to trigger specific actions under a given event (e.g., flooding prediction) [49], but not in relation to having partitions for different purposes.

Finally, Carnevale [50], Villari [51] et al suggest an interesting approach about using osmotic computing to facilitate dynamic resource allocation in IoT environments which would be interesting to consider for future enhancements of this IoT end-to-end slicing orchestrator.

## 3. System Design and Implementation

**Figure 2:**
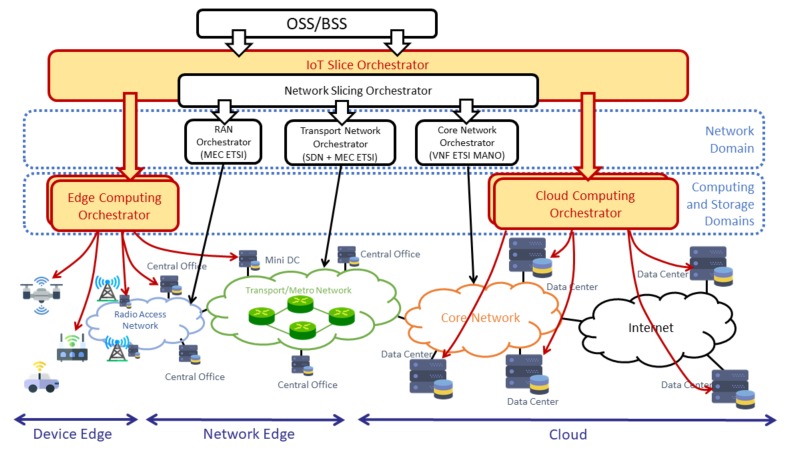
Overall IoT solution orchestration.

The architectural design of the proposed solution is shown in Figure 2. It includes an IoT slice orchestrator as the fundamental component in the design that enables the definition and deployment of IoT slices across multiple domains. These domains encompass radio access networks, aggregation and transport networks core networks, as well as edge/cloud infrastructures. The isolation of end-to-end IoT slices is achieved with the definition of network slices in the network domains and the support of multi-tenancy in the computing and storage infrastructure. With the purpose of creating network slices, the IoT slice orchestrator has an embedded network slice coordinator function (Network Slicing Orchestrator in Figure 2), which coordinates the operation of the diverse network orchestrators that operate locally at each network domain.

The orchestrator is the functional entity in charge of coordinating the operations related to the deployment and configuration of the software pieces (in our case network functions) composing an IoT service, as well as and the connectivity among them. It provides an interface to the Operations Support System/Business Support System (OSS/BSS), which enables the latter to request the creation of IoT slices with specific requirements. These requirements are not only specified in terms of computing, storage, and network resources. They are also related with the specific locations (e.g., edge and/or cloud domains and transport network) where these resources should be provided.

For instance, if a given IoT service requires the delivery of information with low latency constraints, the OSS may request the IoT slice orchestrator to create an IoT slice and deploy some of the functions needed to provide the service at the edge domain. The orchestrator then creates these functions at the proper locations (either at the edge or in the cloud), and creates the appropriate network slices in the transport network, connecting the devices and the functions, and considering for instance whether NB-IoT, LTE-M or GSM [52] are the right required radio access technologies to be used.

We want to highlight that, given the multiple aspects concerning the end-to-end orchestration of IoT slices, this paper has a main focus on the specific functionalities and mechanisms that must be provided by the orchestrator to set up the slices. The orchestrator design enables the establishment of appropriate network slices, the definition of edge computing and cloud computing tenants, and the deployment of the corresponding IoT functions. The service logic related with decision making is still a matter of further discussion and development. This logic could be provided by an internal module of the orchestrator, which could take decisions regarding the placement of functions and the utilization of specific transport networks aiming at optimizing specific parameters of the IoT service (e.g., reducing latency). Alternatively, the placement decisions and the selection of transport networks can be provided to the orchestrator as a set of business rules, generated by an Operations Support System/Business Support System (OSS/BSS).

Once an end-to-end slice is created, the IoT slice orchestrator deploys and/or configures these required functions in the required location. For that reason, the IoT slice orchestrator is integrated with other orchestrators specialized in deploying and configuring functions in a part of the network. In this paper, we make the general assumption that different domains (cloud, edge, and transport networks) may be owned by different providers. These providers establish service level agreements with the IoT service provider, who coordinates the creation of end-to-end slices for the diverse IoT services through the orchestrator. Therefore, cloud/edge domain and transport providers do not have to exchange management information among them, only with the IoT service provider. The implementation of the interfaces consumed from these domain orchestrators will depend on the technology used to build them and the service level agreements established among the different providers.

The IoT slice orchestrator shall handle the following entities, making use of interfaces provided by these other orchestrators:

- The IoT gateway is deployed and configured using an edge or a cloud computing orchestrator, depending on where the gateway is finally deployed.
- The network slice at the access network, configuring the right Radio Access Technology (RAT) and reserving the required resources (e.g., radio channels).
- The network slice at the transport network, for instance using an SDN controller that defines the corresponding Virtual Private Network (VPN).
- The network slice in the core network, deploying or configuring the required Virtual Network Functions (VNFs) to provide this service (e.g., a specific User Plane Function (UPF) or Packet Data Network (PDN) gateway as defined by 3GPP [53] standardization body).
- The IoT server, analytics applications or other functions that are deployed in the cloud, using the corresponding cloud orchestrator.

We want to observe that the development of IoT slice orchestrator is an ambitious work that requires the utilization of diverse 5G-related technologies across multiple domains. For this reason, this paper focuses on the multi-tenancy aspects at the edge and cloud computing domains, which are fundamental in IoT solutions to support the collection, evaluation, storage, and processing of IoT data. Consequently, the implementation of our design, as it is described in later sections, has focused on the IoT gateway and the IoT functions that can be deployed at the edge or at the cloud (i.e., first and last points in the list above). Even the support of network slicing is considered in the design of the orchestrator, as the implementation of the network related part is left for future work.

### 3.1. Orchestration Information Model

**Figure 3:**
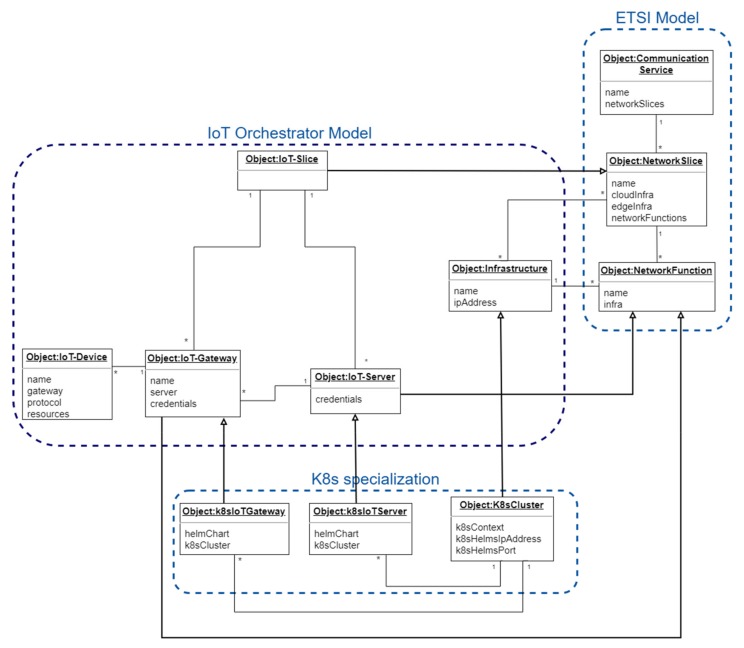
IoT slice orchestrator information model.

In order to support the proper end-to-end orchestration of an IoT service, the information model shown as a UML diagram in Figure 3 has been defined. It represents the internal concepts associated to the orchestration, and the relationships, constraints and rules associated to these concepts [54].

This information model is based on the functional model defined by oneM2M standardization in its functional architecture technical specification [44], the network slice information model defined by the ETSI [55] and 3GPP [56] standardization bodies and on the IoT resource model described by the HINC project [46]. These are the most important entities within the IoT slice orchestrator information model, including concrete information on the standards and concepts used to define them:

(1) The IoT-Device represents the information related to physical or virtual devices with sensing or actuation capabilities; that is, the configuration needed by sensors to provide measurements and by actuators to execute the proper actions. This information is directly mapped to the M2M device in or physical IoT resource in described by M2M [44] and HINC [46] models respectively. The data associated to the IoT-Device entity comprises the protocols used between the devices and the gateway (like MQTT [57], HTTP or CoAP [58]); the magnitude measured by the associated sensors (e.g., temperature or humidity); the address of the IoT-Gateway towards the IoT-Device will be connected; and, in the future, the actions to be executed by the associated actuators.
(2) The IoT-Gateway includes the data associated to a gateway function deployed in an IoT intermediate node (i.e., M2M gateway in M2M model [44] or software defined gateway as proposed by the HINC project [46]) and that could be located at the edge of the network as specified by ETSI in one of its edge scenarios [59] or in the cloud. The information to be configured in those cases is the associated slice, the physical infrastructure where they are deployed (e.g., edge node) and the IoT server connected to this gateway.
(3) The IoT-Server defines a virtualized M2M application [44] or cloud service [46] entity deployed in a network node that runs the business logic and whose main purpose is to collect data from IoT devices and to post-process it for its consumption by other applications like analytics functions (the IoT server may hold these functions). The information contained in this entity is the associated slice, the physical infrastructure (e.g., data center) where it is deployed, and the credentials used to connect to this server.
(4) The IoT-Slice represents an end-to-end network slice [56], that is, a section or a partition of an end-to-end IoT application dedicated to a particular purpose (generally to a specific vertical or customer), including the networking part connecting the entities composing this IoT-Slice. Virtualization techniques (computation virtualization and network virtualization) are used to implement this concept. The information associated to this entity consists of the identifiers of the data centers (infrastructures) where this slice is implemented.
(5) The Infrastructure refers to the location where the virtual functions are deployed. Typically, there will be locations at the edge, at some small offices in the middle of the network or in a data-center in the cloud. In the model shown in Figure 3, the data associated to this entity is an IP address to access the infrastructure. Real uses cases may extend this entity definition with additional fields, as needed.
(6) Communication Service [55,56] represents the abstraction of a service offered in a telecommunication context that in the case of IoT it can be for instance an analytics service for IoT devices.
(7) The Network Slice represents a list of network functions (including their resources) connected and arranged so that they can provide a logical network for a specific purpose. The proposed IoT slice is a specialization of a network slice containing specific IoT network functions (e.g., IoT gateway).
(8) Network Function is an entity with some functional behavior provided by some external interfaces. These network functions can be virtual (VNF) or physical (PNF) and are deployed at the edge, in the middle of the network or in a cloud data center. The proposed IoT gateway and server are particularizations of network functions

As it will be seen in the implementation section, containerized applications have been selected. The open-source Kubernetes orchestration platform [20] is used to deploy the containers and Helm [60] to pack and install these applications in a Kubernetes cluster. For this reason, some of the abstractions in the IoT slice orchestrator information model (like IoT Gateway, IoT Server and Infrastructure) are extended with some additional attributes related to a Kubernetes deployment:

- The infrastructure is specialized with some Kubernetes cluster information like Helm related data and the context information needed by the orchestrator to configure and connect to this cluster.
- IoT-Gateway and IoT-Server entities are specialized with the data related to the Helm charts used to install these containerized applications in the Kubernetes cluster.

### 3.2. Implementation

The IoT gateway (Edge X Foundry [61]) and IoT server (Mainflux [62]) functions used in the validation environment are implemented using microservice-based architectures. In relation to the orchestration of the IoT functions, the REST interface offered by Kubernetes [20] is used to orchestrate the microservices composing these functions.

As it was stated before, the IoT slice orchestrator that has been designed to be able to deploy IoT servers and IoT gateways in different locations (edge and cloud). From the implementation perspective, this is done through the command line interface described in Annex A and implementing the information model described in Section 3.1. In this paper, those locations are obviously Kubernetes clusters [20], but it could be any other type of infrastructure for virtual applications. For the microservice installation and initial configuration, Helm [60] is used in both cloud and edge environments.

It is important to highlight that the Kubernetes cluster does not introduce a significant load that requires powerful and heavy equipment. On the contrary, it can be deployed in constrained devices like Raspberry Pis, which are single board computers with limited computing and storage capacity using Wi-Fi as radio access technology. In fact, this edge environment is configured in several Raspberry Pis (it can even be installed just in one) which could be easily assembled in a SUAV device, in a similar way as it is done by Nogales et al in [63]. Therefore, with the orchestrator, it is possible to deploy the IoT gateway in a Raspberry Pi installed in a SUAV device and connect it to an IoT server in a few seconds, even when it is in the air.

**Figure 4:**
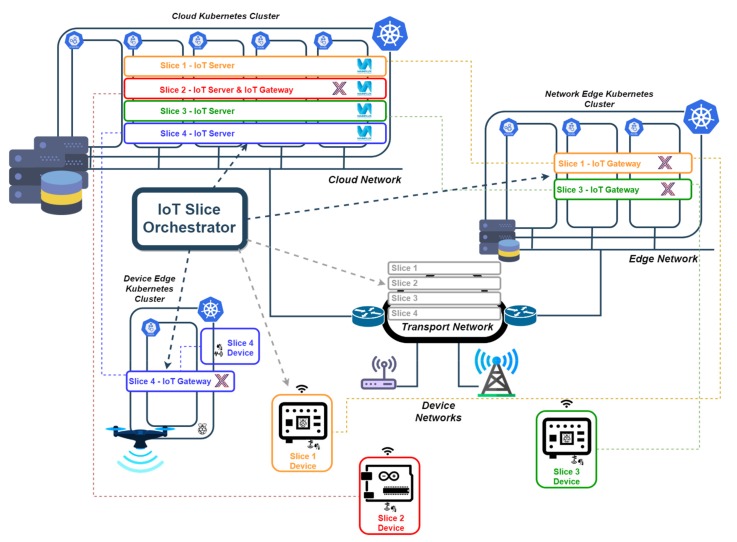
Example of deployment of IoT end-to-end architecture using edge and cloud computing; and network slicing.

Depending on the requirements, the IoT gateway (or even the IoT server) are deployed either at the edge cluster (at the network edge or at the device edge) or in the cloud Kubernetes cluster. In Figure 4 there is an example where the IoT slice orchestrator creates four slices, where one is entirely created in the cloud (slice 2), two where IoT gateway is deployed at the network edge (slices 1 and 3) and another one where the gateway is installed at the device edge assembled to a SUAV device.

The IoT slice orchestrator also ensures that the IoT gateway and the IoT server are connected. Nowadays, the underlying connectivity between the pieces of the solution (IoT device to IoT gateway and IoT gateway to IoT server) is assumed to be manually done by configuring the needed routing, a L3VPN [64], a GRE tunnel [65] or a VxLAN tunnel [66], for instance. However, in the next phases of the investigation, the IoT slice orchestrator shall be able to configure the connectivity using an SDN controller. Further implementations of the orchestrator will allow the system to integrate with 5G networks, being able to define network slices or making use of pre-established network slices.

In Figure 4, IoT devices are represented by sensors connected to Raspberry Pis or Arduino boards, which are also single board computers. However, the described system does not preclude from having other more constrained devices and other access technologies like Bluetooth Low Energy [67], ZigBee [5] or any cellular IoT technology (e.g., NB-IoT) [52].

## 4. Validation of the Solution

This section, describes the details of the solution, including the open source software that has been used, the updates on this software, the insights of the developed prototype and the environment used to validate it.

The test environment built to execute this validation is a simplified version of the deployment example shown in Figure 4 using only two slices and a subset of the equipment included in this example. The validation is performed with the execution of some functional tests to prove the functions deployed with the orchestrator work with real IoT devices, and the execution of some performance tests to confirm that the orchestration is able to deploy functions at the edge and in the cloud and they work as expected, but not as a benchmark exercise.

### 4.1. Server and Gateway Software

For the implementation of the IoT gateway and the IoT server, Edge X Foundry framework [61] and Mainflux IoT platform [62] have been respectively used. Both of them are open-source functions.

As it was indicated in the previous section, these implementations use microservice-based architectures that offer huge flexibility at deployment time. In fact, we have orchestrated the minimum set of microservices (and their replicas) were orchestrated for both applications, in order to have a minimum functionality with minimum computation and storage requirements.

Nevertheless, some adaptations were needed to support this validation scenario, specially to allow their integration in Kubernetes. These updates are included in Table 1 and can be found in [68,69,70,71].

More details about the internal microservice architecture of these two applications can be found in Appendix B and Appendix C.

### 4.2. Orchestration Software

Python programming language [73] has been used to implement the IoT slice orchestrator [74] following the model shown in Figure 3. It provides a Common Line Interface (CLI) that offers the following operations:

- Creation, removal, retrieval, and list of Kubernetes clusters where the IoT gateway and server are deployed.
- Creation, removal, retrieval, and list of IoT slices associated to an edge and a cloud Kubernetes cluster.
- Creation, removal, retrieval, update, and list of IoT servers.
- Creation, removal, retrieval, update (attach to IoT server), and list of IoT gateways.
- Creation, removal, retrieval, and list of IoT devices attached to an IoT gateway.

As it was stated in previous sections, the IoT slice orchestrator integrates with edge and cloud orchestrators (in our prototype they are Helm charts for Kubernetes deployments). In order to support this possibility, the orchestration software makes use of the existing Python libraries (PyHelm [75] and Kubernetes Python client [76]). More details about the internal implementation of the orchestrator can be found in Appendix A.

### 4.3. Protocol and Message Formatting

It is important to remark that IoT solutions may be diverse, considering: the heterogeneity of sensors and actuators; the vast variety of devices onboarding those sensors, which may present different characteristics (e.g., memory or processing capacity); the different radio access technologies supported by these devices (e.g., Bluetooth or Narrowband IoT); and the considerable number of open-source and commercial software platforms providing IoT-related functions which are able to collect and analyze all these measurements. However, there is still one important aspect to be considered, and it is related to the amount of protocols used in IoT solutions and the format used to send the measurements obtained from the sensor.

In relation to the protocol, MQTT [57] has been used, since it was already supported by both Edge X Foundry framework and Mainflux platform and because there is a wide range of available devices and libraries providing this protocol.

Regarding message formatting, the Sensor Measurement Lists (SenML) format has been used. It is defined in an already standardized IETF specification [72] which was already supported by Mainflux platform. Edge X Foundry software was updated to support this format [70,71] and specific Python clients were implemented to send SenML formatted data over MQTT from any device supporting Python [77].

### 4.4. Test Environment

In order to do the validation of the IoT slice orchestrator various Kubernetes clusters and distinct types of devices have been used and they are described in this section. Regarding Kubernetes clusters, three different clusters have been used:

- A three Kubernetes node cluster deployed in Google Compute Engine, with two virtual CPUs and 7.5 gigabytes of memory per node. A Network File System (NFS) server with 10 GB of disk in the Kubernetes cluster had to be deployed since Google Compute Engine Persistent Disks do not support writing operations from different containers and it is required by the Edge X Foundry framework. In this cluster two instances of Mainflux (two IoT slices) were deployed and one instance of Edge X Foundry (for one slice). This cluster was used to compare the latency and throughput figures when the IoT gateway is deployed at the edge and when it is deployed in the cloud.
- Another cluster composed by three Raspberry Pis 3B+ (cluster could have been created even in one board), all of them with one CPU core and 1 GB of RAM. The storage used in this case is provided through the file system of the Raspberry Pis. In this environment only one instance of the Edge X gateway was deployed for one of the slices. This cluster was also used to compare the latency and throughput obtained when IoT gateway is deployed at the edge and when it is deployed in the cloud. It is important to note that the number of Raspberry Pis in this cluster can be increased or reduced, according to the requirements of the IoT service to be deployed. In addition, the cluster can easily be onboarded on mobile units, such as small unmanned aerial vehicles, following the approach described by Nogales et al in [63] where a Raspberry Pi board is assembled in this type of vehicles. This enables a cost-effective solution to offer the functionality of an IoT gateway in IoT scenarios where network infrastructures are insufficient (or simply unavailable), to support data communications with IoT devices over a delimited geographic area (e.g., a remote area in smart farming applications).
- Finally, one custom Kubernetes cluster virtualized in four virtual machines (each one with 2 virtual cores and 4 GB of RAM) created in a server with an 8 core AMD processor and 32 GB of RAM. The storage used in this case is represented by the file system of the server. This environment provided a baseline platform to evaluate the time used by the IoT slice orchestrator to create and deploy one IoT slice and to verify that several slices can cohabitate and properly operate within the same cluster (see Appendix D). In this case, both an instance of the IoT Gateway and an instance of the IoT Server were executed on the same server for each slice.

**Figure 5:**
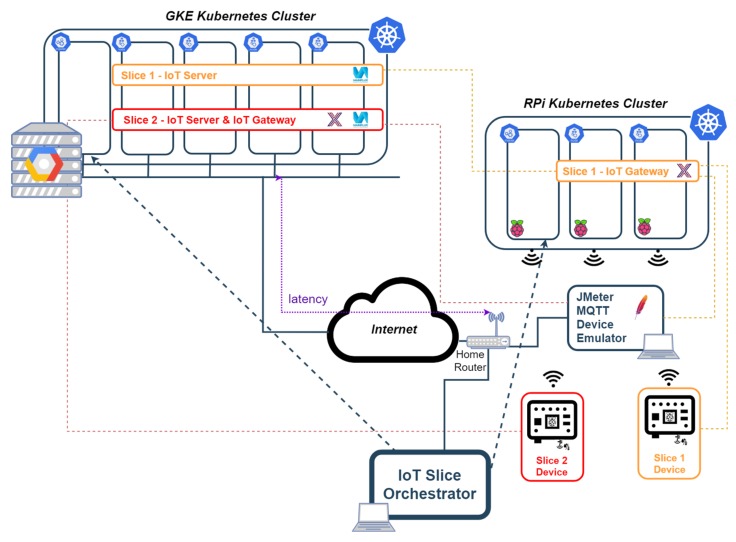
Test environment.

Figure 5 shows the test scenario that has been built to perform the functional validation of the solution. This scenario uses the first two clusters in the list above, and it is as a simplified version of the example shown in Section 3.2, considering only two slices, which is sufficient for functional validation purposes. This setup is also used to compare performance figures in terms of end-to-end latency and in terms of throughput.

In one of the slices (slice 1), an IoT gateway is deployed in a Raspberry Pi of the second Kubernetes cluster above, while the IoT server is deployed in Google cloud; in another slice (slice 2), both the gateway and the server have been deployed in google cloud (first cluster in the list above). Both clusters are interconnected through the Internet, using a commercial router that is able to route messages from the internal laboratory network, where the Raspberry Pi cluster is connected, to the Google Cloud, and vice versa.

Two types of devices have been used to perform the validation:

- Real devices like Raspberry Pis (model 3B and model Zero) [78] and Arduino Yun boards [79] connected to a set of sensors widely used in connected home environments to measure temperature, humidity and barometric pressure (DHT11 [80], DHT22 [81], BMP180 [82], BMP280 [83] and BME280 [84]). As stated before, the code executed in Raspberry Pis and Arduino Yun board is based on Python [73, 77]. Those devices are used to validate the correct operation of the solution in a real setup.
- Emulated devices using JMeter Apache load tester application [85] with an MQTT plug-in [86] configured to send SenML formatted random temperatures. This application is used to send traffic to validate that the solution works and to stress the system in order to obtain latency and throughput figures.

Apart from the IoT gateway and the IoT server installed in the Kubernetes clusters, some other container-based applications are also deployed in order to obtain the measurements that are used to validate the solution. Some of those measurements are shown in next section. InfluxDB open-source time series database [87] has been deployed together with the IoT gateway and the IoT server, and also Grafana, an open-source platform for time-series analytics [88]. Although both Edge X gateway and Mainflux server already supported the integration with InfluxDB for analytics, some small updates on the existing code were required [68,70] as it was shown in Table 1.

In relation to the connectivity of the environment, two Ethernet LANs and one wireless LAN (WLAN) were installed in the laboratory. The wireless LAN supports the communications between the Raspberry Pi cluster and the sensor devices (needed in slice 1); and between the sensor devices and the commercial routers (needed in slice 2). The two Ethernet LANs connect, respectively: the router and the laptop where the IoT slice orchestrator is running; and the router with the computer where the JMeter device emulator is executed.

### 4.5. Functional Validation Using Real Devices

For the functional validation, real devices are used to measure the temperature in different places of a particular house using multiple sensors (two Raspberry Pis model 3B with a BME280 sensor attached and one Raspberry Pi Zero with a DHT22 sensor attached). In the background, some random temperatures are also sent from JMeter load test application. When testing the slice with the IoT gateway deployed in the Raspberry Pi Kubernetes cluster (slice 1), all these devices are connected to this IoT gateway, and when testing the other slice (slice 2), all of them are connected to the IoT gateway deployed in Google Kubernetes Engine (GKE) [89].

**Figure 6:**
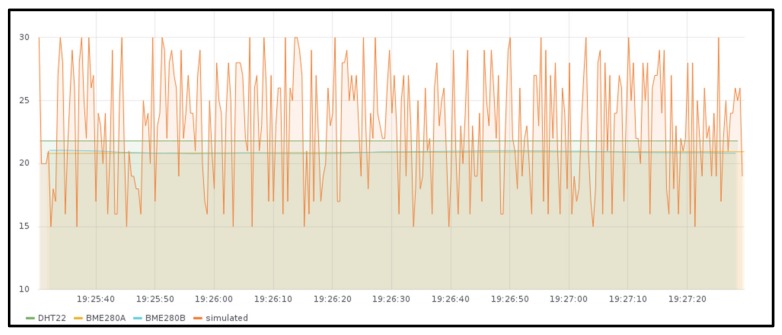
Temperature generated by BME280 and DHT22 sensors together with emulated background traffic measured at the IoT gateway deployed in Raspberry Pi Kubernetes cluster.

**Figure 7:**
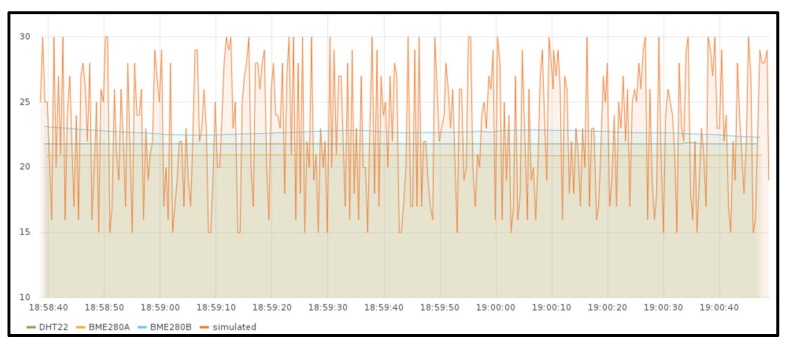
Temperature generated by BME280 and DHT22 sensors together with some emulated background traffic measured at the IoT gateway deployed in Google Kubernetes Engine cluster.

Figure 6 shows the measured values stored in the InfluxDB instance deployed together with the Edge X gateway in the Raspberry Pi cluster (slice 1) and Figure 7 shows the ones obtained from the GKE cluster (slice 2), both for around two minutes. As it can be observed, temperature sent from real sensors is steady, while the temperature received from JMeter has fluctuations. There is no difference in the behavior when deploying the gateway in one location or the other, except for the measured values that were obtained at different times.

The measured values obtained in the InfluxDB instance deployed with the Mainflux server in the GKE cluster (slice 1) are not included since the information measured is exactly the same as the one measured in the IoT gateway (Figure 6 and Figure 7).

### 4.6. Latency Measurements

For the latency measurements, the test environment described in Figure 5 is used as well. The time required to process the readings by both the gateway and the server is negligible compared to the latency introduced by the network, and particularly to the one introduced between the home router and the Google Compute Engine. Hence, for latency calculation the time between the reading taken at the IoT gateway and the reading taken in the IoT server will be compared for the same temperature measurement for each slice (i.e., when the IoT gateway is deployed in the Raspberry Pi cluster, slice 1, and when it is installed in the GKE cluster, slice 2). In this case only emulated devices using JMeter load test tool connected to both slices are used. It is manually ensured that the Raspberry Pi cluster uses the same NTP [90] server as GKE cluster and time is forced to be synchronized just before the testing.

**Figure 8:**
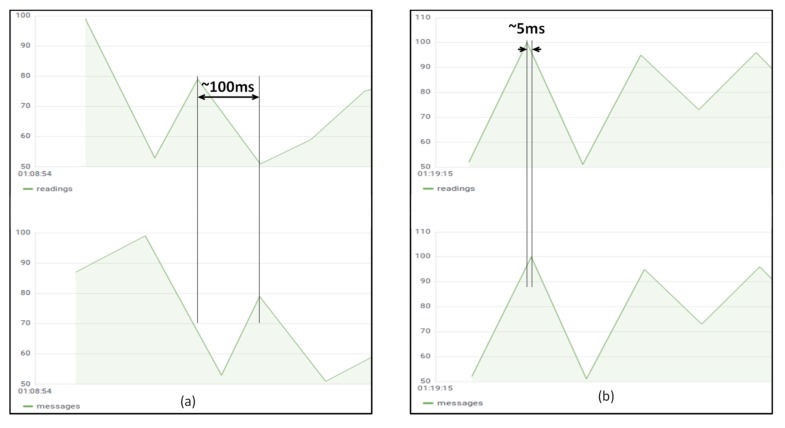
Latency measured between the IoT gateway and the IoT server when the gateway is executed in the Raspberry Pi Kubernetes cluster (**a**) and when the gateway is executed in the Google Kubernetes Engine cluster (**b**).

Latency measurements change considerably depending on the impairments of the network at the moment of taking the measure. Figure 8 shows a situation where the network latency measured between the home router and the GKE cluster is around 100 milliseconds.

As it can be observed, the latency when deploying both the gateway and the server in GKE is much smaller (around 5 milliseconds in Figure 8b) than when the gateway is executed in the Raspberry Pi cluster, that is around the latency introduced by the network connecting the IoT gateway and the IoT server (100 milliseconds in Figure 8a).

Obviously, the deployment of the IoT gateway at the edge is always a good choice when trying to execute some pre-processing activities in the gateway since it reduces the bandwidth between the gateway and the server. However, with these tests it is also possible to prove that deploying at the edge is better for applications with significant latency requirements. For instance, if a fast reaction on an actuator connected to the device is needed, as a result of a measurement obtained from a nearby sensor (e.g., onboarded at the same device), this reaction is executed faster if this trigger is accomplished in the gateway deployed at the edge (slice 1).

### 4.7. Throughput Measurements

Before describing the tests executed to measure the throughput obtained when deploying the Edge X gateway in the Raspberry Pi cluster (edge), and when it is deployed in the GKE (cloud), it is important to mention that these tests do not aim to provide benchmark figures or a possible comparison between edge and cloud computing. Performance metrics would typically depend on the IoT gateway and the IoT server implementations, which are under development in their respective open-source communities at this moment. The objective of these tests is to verify which is the best deployment option (edge or cloud) for the IoT gateway, when throughput requirements are more relevant than latency related requirements in an IoT solution. This will typically depend on the capacity that can be made available by edge-computing platforms (in our tests, edge nodes consist in a Kubernetes cluster made of several constrained devices). Throughput testing was also performed using the environment shown in Figure 5 with emulated devices using JMeter. Now, the comparison is done between the throughput measured when allocating the IoT gateway at the edge (slice 1) or in the cloud (slice 2). In this case, temperature measurements are not considered, just the number of hits per second in all InfluxDB time series databases. 

When the gateway is executed in the Raspberry Pi cluster (slice 1), several trials were performed, and the maximum throughput obtained was around 10 hits per second and was decreasing when the sending rate was higher than this number.

**Figure 9:**
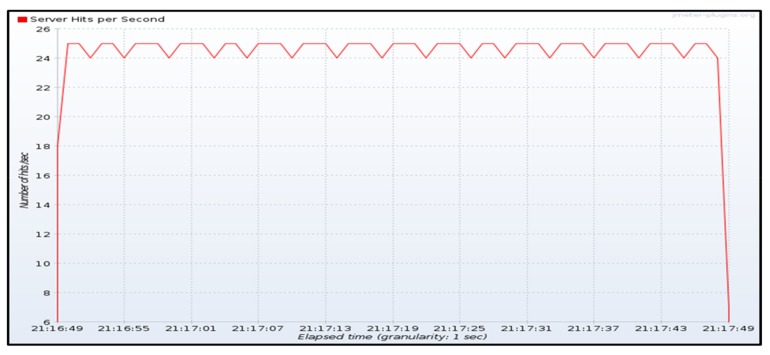
Throughput measured at JMeter source (~24 hits/second).

**Figure 10:**
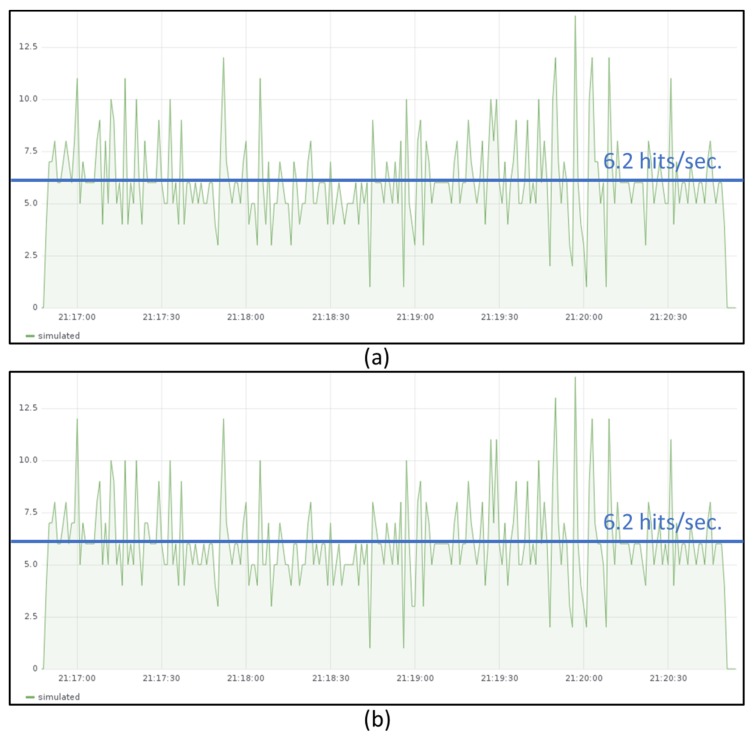
Throughput measured at the IoT gateway (**a**) and the IoT server (**b**) when the gateway is deployed in the Raspberry Pi cluster.

Figure 9 shows the transmission of temperature measurements during one minute from JMeter emulated devices with a sending rate of around 24 messages per second. Figure 10a shows the throughput measured in the IoT gateway and Figure 10b the one measured in the IoT server. The throughput obtained in both sites is around 6.2 temperature hits per second and the time required to process all received messages is almost four times the sending period. Those measures also show that the limiting function is the IoT gateway since IoT server is able to follow the rate generated by the gateway.

Some measurements were also performed both at the IoT gateway and at the IoT server with different rates when the IoT gateway is executed in GKE cluster (slice 2). In this case the maximum throughput obtained is around 18 hits per second and the throughput figure is not decreasing so drastically as it was observed when the IoT gateway was deployed in the Raspberry Pi cluster.

**Figure 11:**
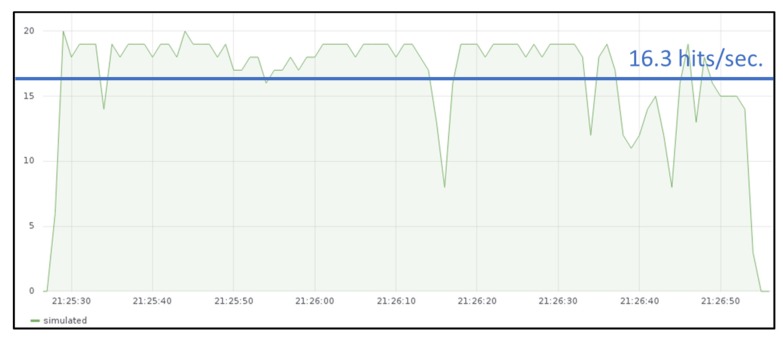
Throughput measured at IoT server when gateway is deployed in GKE cluster.

When sending messages from JMeter emulated devices with a sending rate of around 24 messages per second (similarly as it was shown in Figure 9), the throughput measured in the IoT gateway is around 16.3 temperature hits per second (Figure 11). Hence, Edge X gateway has almost three times better throughput when it is deployed in the cloud.

With the hardware resources allocated to the IoT gateway at the Raspberry Pi, the throughput figures are lower than in the case of IoT gateway deployment in the GKE cluster. This is an expected result in realistic IoT deployments, where edge infrastructure resources may be limited in terms of computing and memory capacity.

It must be highlighted again that the target of this research is not to develop optimum software implementations of the IoT gateway and server in order to obtain high throughput values, as it is expected from a commercial system. Even if this is not part of this research, it is important to mention that one way to increase the throughput could be achieved by changing the system to allow having more than one microservice replica (in this work a minimum set of microservices with just one replica is used), performing an efficient load balancing among these replicas. On the other hand, communication among microservices in the IoT gateway does not seem to be so efficient as in IoT server where a message bus like NATS [91] is used to communicate all microservices. Hence, optimizing internal communications among Edge X components may also increase service performance.

## 5. Conclusions

This article has shown the relevance of an appropriate end-to-end coordination of resources to support practical IoT deployments. A design proposal for an IoT slice orchestrator has been presented. This orchestrator enables the cohabitation of multiple instances of an IoT solution for distinct customers, over a set of shared infrastructures. The proposed design leverages the capacity of orchestrating and managing the network slices of 5G networks [56], complementing it with the automated deployment of other specific IoT related functions, like an IoT gateway or an IoT analytics server. As a first step to prove the practical feasibility of our work, we have focused on the multi-tenancy aspects at the edge and cloud computing domains. In this respect, we have implemented a functional prototype of our design, supporting end-to-end orchestration procedures on a real environment, using a commercial cloud platform, i.e., Google Compute Engine, as well as an open-source microservice platform, i.e., Kubernetes, which serves to provide functions at the edge of the network.

The prototype implementation has been used to validate the capacity of our design to support the deployment of the pieces composing an IoT slice (e.g., IoT gateway) at different network locations (at the edge and at the cloud), even on resource-constrained single board devices that can be available at the edge with very limited cost (for instance transported over SUAV platforms). In this respect, the paper evaluates the benefits of this approach in terms of latency using a specific experimental setup. As a result of this work, we have also made several contributions to the open-source community, which are summarized in Table 1 in Section 4.1.

Our future work will cover the networking aspects that are relevant to end-to-end orchestration, particularly those related with the creation of network slices over the radio access, transport and core networks of telecommunication operators. In addition, we will address a detailed study of the options to support the decision logic regarding the placement of IoT functions and the selection of specific transport networks, aiming at optimizing specific parameters of the IoT service (e.g., reducing latency)

## Figures and Tables

**Table 1:** Main software updates done on Edge X Foundry and Mainflux platforms.

Platform	Summary of Updates
Edge X Foundry	Support for SenML format [72] over MQTT on incoming messages
Edge X Foundry	Support for SenML format [72] on proxied messages
Edge X Foundry	Updates on timestamp handling for time series storage (InfluxDB)
Edge X Foundry	Integration with Kubernetes
Edge X Foundry	Helm charts definition
Mainflux	Updates on timestamp handling for time series storage (InfluxDB)
Mainflux	Updates on the integration with Kubernetes
Mainflux	Helm charts definition

**Figure A1:**
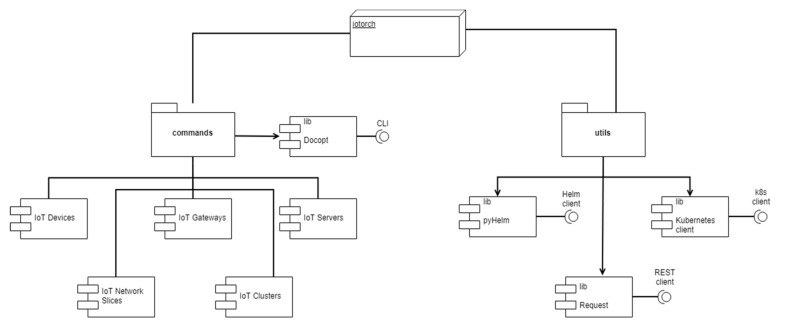
IoT slice orchestrator modular architecture.

**Figure A2:**
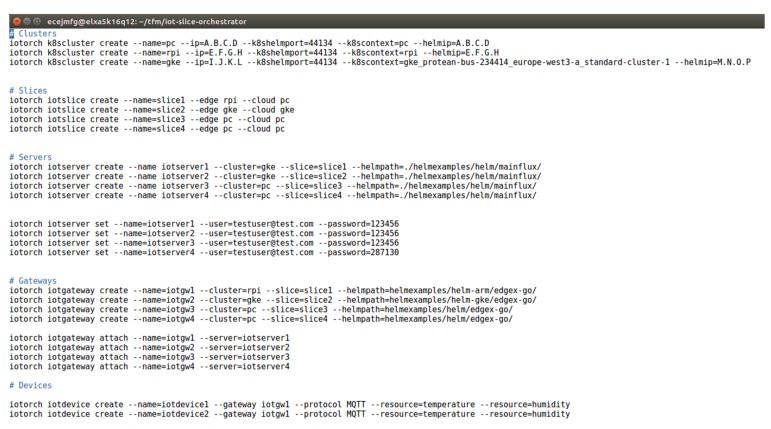
Example of commands used to create slices, servers, gateways and devices

**Figure A3:**
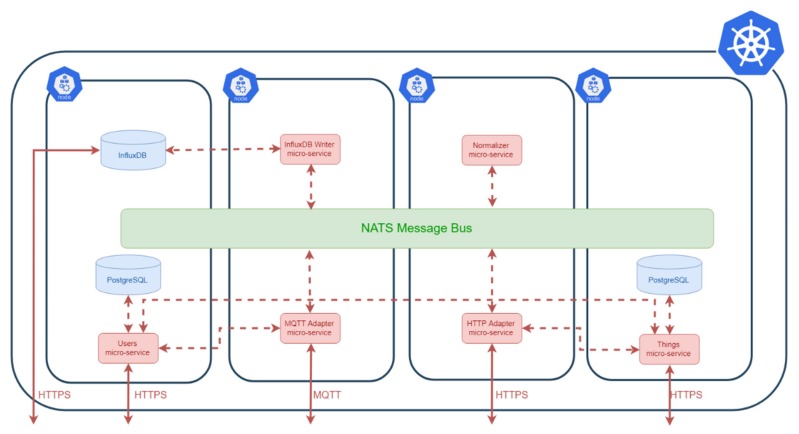
Mainflux microservice architecture.

**Figure A4:**
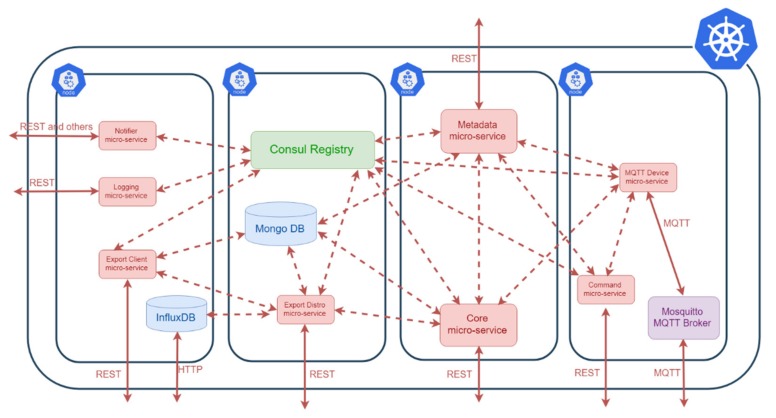
Edge X Foundry microservice architecture.

**Figure A5:**
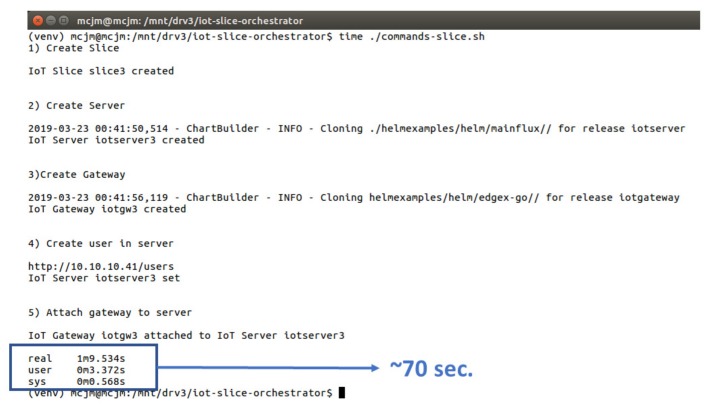
Time measured to deploy an IoT slice with its corresponding IoT gateway and IoT server in a custom Kubernetes cluster.

**Figure A6:**
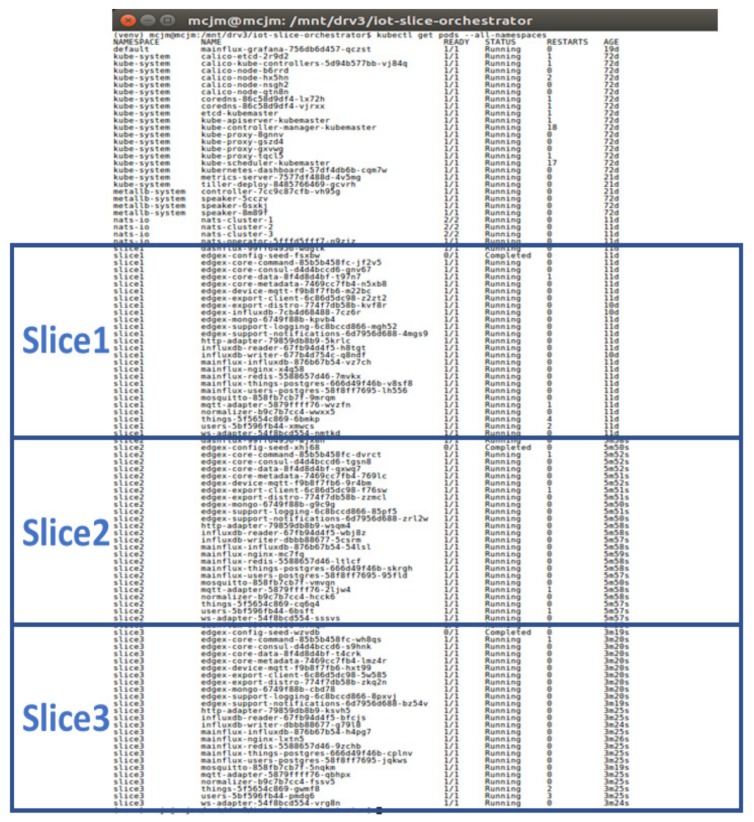
Example of three slices deployed in a custom Kubernetes cluster.

## Appendix A. IoT Slice Orchestrator (Iotorch)

The IoT slice orchestrator [74] has been implemented in Python language following a modular architecture as it is shown in Figure A1. It uses Docopt libraries [92] to implement the commands offered through its command line interface and it uses other Python libraries like PyHelm [75] or Kubernetes Python client [76] to allow the integration with IoT gateway and IoT server orchestrators based on Helm and Kubernetes.

In the future it is intended to change this architecture to introduce plug-ins which will allow the integration of the IoT slice orchestrator with other orchestrators available for other types of edge and cloud platforms.

A list of commands of the IoT slice orchestrator CLI is shown in Figure A2. These commands are used to create the slices used in the validation environment that was shown in previous sections.

## Appendix B. Mainflux IoT Server

Mainflux [62] is an open-source IoT platform written in Go language [93]. It has a microservice-architecture where the pieces composing its business logic are contained in independent microservices. As shown in Figure A3, most of the microservices can communicate among them by the usage of the NATS message bus [91] which is an open-source messaging system for cloud native applications.

The following are the most important Mainflux microservices used in this prototype:

- Users microservice, which allows IoT platform to create a user account that is used to configure the connection between Edge X Foundry IoT gateway to Mainflux server. It its connected to a PostgreSQL database [94] to store all the user information.
- Things microservice, which is in charge of provisioning devices and configure communication channels used by these devices. This microservice is used to create the MQTT topic (channel) from Edge X Foundry IoT gateway to Mainflux server and provision Edge X Foundry IoT gateway as a “Mainflux thing” within the prototype. It its connected to a PostgreSQL database to store the provisioned device information, but not the events.
- Normalizer service, that is responsible for normalizing the events published by the connected things in the SenML format which are finally stored in the events databases.
- MQTT adapter, that includes an MQTT broker listening for events published by the provisioned devices. These devices are mainly the Edge X Foundry IoT gateway in the prototype.
- HTTP adapter, which offers an HTTP API that is used by the devices to send their measurements. There are other adapters for CoAP or WebSocket interfaces, but they are not used by this prototype yet.
- Database writers, which are in charge of storing normalized data into a database which is an InfluxDB time-series database [87] in the prototype.

All these microservices are already containerized [95] and some unofficial guides to deploy it in a Kubernetes cluster are given. In order to facilitate the installation of Mainflux application in a Kubernetes Cluster, specific Helm Charts [68] were created for this prototype.

## Appendix C. Edge X Foundry IoT Gateway

Edge X Foundry framework [61] “is a vendor-neutral open source project hosted by The Linux Foundation building a common open framework for IoT edge computing”. It is implemented in Go language [93] and Java and it is intended for building IoT gateways. Its architecture is also based on microservices which can be containerized. All these microservices can communicate with each other using well-defined interfaces as it is shown in Figure A4.

The following are the most relevant microservices in Edge X Foundry platform [96,97] used in this prototype:

- Configuration and Registry microservice which provides configuration information to the rest of the microservices when they boot-up is the configuration service is implemented using Consul [98]. When it is executed in a Kubernetes cluster, all the rest of microservices wait until this microservice is properly installed.
- Core is the microservice in charge of storing the measurements received from the devices in a MongoDB database [99]. It uses a ZeroMQ messaging queue [100] and publishes the events that are later collected by Export Distribution microservice.
- Metadata microservice offers the REST interface that is used to provision devices. This is the interface used by IoT slice orchestrator to provision device information in Edge X gateway whenever a new device is created. This information is stored in the MongoDB database [99].
- Command microservice will be used in the future to send commands towards the devices (e.g., to send MQTT notifications to trigger an actuator).
- Export Client microservice offers a REST interface to configure external entities. Edge X gateway will forward collected measures to these entities. This is the interface used by IoT slice orchestrator to connect Edge X gateway with Mainflux using MQTT protocol. This interface is also used to send the received measurements to an external InfluxDB time-series database [87] for post-processing. Note this InfluxDB instance is not part of official Edge X Foundry, but it was deployed in the prototype to do the analysis shown in previous sections.
- Export Distribution microservice, which receives the measurement events from Core microservice and forwards them to the configured export clients (i.e., Mainflux and InfluxDB). This microservice was updated [70] to support forwarding measurements in SenML format [72].
- MQTT Device Service, which is an MQTT client able to receive an generate MQTT publish and subscribe messages. This microservice is connected to a configured MQTT broker; it subscribes to the MQTT topic used by the devices and it receives messages published on this topic. In the future it will also publish MQTT messages that will trigger some event in an actuator. This microservice was updated to support receiving measurements in SenML format [72].
- Edge X Foundry platform relies on an external MQTT Broker; however, it is important that this broker is also part of the IoT Gateway in the prototype. For that reason, a container with a lightweight MQTT broker, the Mosquitto MQTT server, [101] is deployed. This broker receives MQTT publish messages from the devices and forwards them to MQTT Device microservice.

All these microservices were already containerized, however, specific Docker images were built to allow its execution on Raspberry Pi. Edge X Foundry platform did not have any support for Kubernetes, so this support including Helm charts were created from scratch [69].

## Appendix D. Service Creation Time

For the execution of these tests, the Kubernetes cluster virtualized in four virtual machines, as described in previous sections, was used. The execution times vary depending on different aspects such as the computing capacity of the system (e.g., type of processors and memory), the storage used since time to allocate this storage might take longer depending on the infrastructure, and the external IP allocation which can take some time depending on the system (which usually takes longer in commercial platforms like GKE). However, it is interesting to see the order of magnitude of this execution time.

A specific script was designed to create an IoT slice in the cluster, deploy Edge X gateway, deploy Mainflux server and connect both gateway and server through the orchestrator command line interface. The result of this experiment showed appropriate values for the service creation time as it is shown in Figure A5; the orchestrator is capable of deploying and configuring an IoT service in a bit more time than one minute.

Finally, and in order to show that it is possible to deploy multiple slices within the same infrastructure, Figure A6 illustrates the deployment of three slices with the corresponding Edge X gateway and Mainflux server in the Kubernetes cluster virtualized in four virtual machines. Note the status of all relevant Kubernetes PODs is “running”.
